# Effect of Medium Supplements on *Agrobacterium rhizogenes* Mediated Hairy Root Induction from the Callus Tissues of *Camellia sinensis* var. *sinensis*

**DOI:** 10.3390/ijms17071132

**Published:** 2016-07-15

**Authors:** Mohammad M. Rana, Zhuo-Xiao Han, Da-Peng Song, Guo-Feng Liu, Da-Xiang Li, Xiao-Chun Wan, Alagarsamy Karthikeyan, Shu Wei

**Affiliations:** 1 State Key Laboratory of Tea Plant Biology and Utilization, Anhui Agricultural University, 130 Changjiang Blvd West, Hefei 230036, China; ranabtri@yahoo.com (M.M.R.); hanzx1226@163.com (Z.-X.H.); sdp20073882@163.com (D.-P.S.); babcule1989@163.com (G.-F.L.); dxli@ahau.edu.cn (D.-X.L.); xcwan@ahau.edu.cn (X.-C.W.); mdukarthimicro@yahoo.com (A.K.); 2 Agronomy Division, Bangladesh Tea Research Institute, Srimangal-3210, Moulvibazar, Bangladesh

**Keywords:** *Agrobacterium rhizogenes*, antioxidants, browning, callus, hairy root, tea

## Abstract

Tea (*Camellia sinensis* L.) is recalcitrant to *Agrobacterium*-mediated genetic transformation largely due to the bactericidal effects of tea polyphenols and phenolics oxidation induced by necrosis of explant tissue over the process of transformation. In this study, different antioxidants/adsorbents were added as supplements to the co-cultivation and post co-cultivation media to overcome these problems for the transformation improvement. Tea-cotyledon-derived calli were used as explants and *Agrobacterium rhizognes* strain ATCC 15834 was used as a mediator. Results showed that *Agrobacterium* growth, virulence (*vir*) gene expression and browning of explant tissue were greatly influenced by different supplements. Murashige and Skoog (MS) basal salts medium supplemented with 30 g·L^−1^ sucrose, 0.1 g·L^−1^ l-glutamine and 5 g·L^−1^ polyvinylpolypyrrolidone (PVPP) as co-cultivation and post co-cultivation media could maintain these parameters better that ultimately led to significant improvement of hairy root generation efficiency compared to that in the control (MS + 30 g·L^−1^ sucrose). Additionally, the reporter genes β-glucuronidase (*gusA*) and cyan fluorescent protein (*cfp*) were also stably expressed in the transgenic hairy roots. Our study would be helpful in establishing a feasible approach for tea biological studies and genetic improvement of tea varieties.

## 1. Introduction

Tea (*Camellia sinensis* L.) is one of the most important beverage crops in the world [1,2]. Tea plants, containing high amounts of bactericidal polyphenols, are generally considered recalcitrant to *Agrobacterium*-mediated transformation [3,4,5]. Recalcitrance of tea explants to *Agrobacterium*-mediated genetic transformation is also closely related to the browning/necrosis of explant tissue caused by toxic compounds derived from oxidation of the explant-released phenolics [1,4,5]. Quinones, often readily formed due to the oxidation of tannin and polyphenols following wounding or stress, are highly reactive and toxic to the plant tissues and *Agrobacterium* [4,6]. Sandal et al. [3] proposed that the following three general requirements should be met for successful tea genetic transformation: (i) the bactericidal effect of polyphenols released from the tea explants has to be overcome; (ii) *Agrobacterium* virulence (*vir*) gene expression must occur; and (iii) the transformed tissues must retain their potential for regeneration.

Plants of *Camellia sinensis* are well known for their high but varying abundances of characteristic polyphenols in different tissues and in different growth conditions [7], and browning is a malady of tea tissue culture due to the exudation of polyphenols [8]. Compared to tea leaves, polyphenols are less abundant in calli [5], although the phenolic content of calli may increase gradually over sub-culturing [9] and phenolic induction by various stress conditions such as application of antiseptics/disinfectants, explant excision and wounding, antibiotic selection, light exposure, and *Agrobacterium* infection [6,10]. Moreover, in the process of *Agrobacterium* infection and co-cultivation of tea explants, chemicals such as polyphenols that are dynamically released from the explant wounding sites would act on *Agrobacterium* cells locally due to their bactericidal effects [11,12]. Song et al. [5] reported that tea catechins significantly reduced both transient and stable transformation events, which is likely due to the suppression of *Agrobacterium* growth. Therefore, sufficient *Agrobacterium* growth on tea explant tissue is required at the co-culture stage. Generally, co-cultivation for two or three days is routinely practiced for most of the crops, but for tea plants an extended co-cultivation period of five to six days is recommended for higher transformation efficiency [13,14], which might also favor explant browning [15]. In addition to optimal *Agrobacterium* growth, activation of *Agrobacterium* *vir* genes such as *virG*, *virB*, and *virD* through *vir*A by the plant-produced phenolic inducers such as acetosyringone [16] is crucial for successful *Agrobacterium*-mediated plant transformation [17] once explant tissue browning is well controlled.

Great efforts have been made to mitigate explant browning in tissue culture for different plant species [18,19,20,21,22]. Reduction in culture medium nutritional elements was also reported to reduce explant browning of *Olea europaea* [19]. Pretreating explants and/or amending culture media with compounds specifically selected to reduce tissue browning are also often employed [18]. Most of these treatments/amendments can be divided into two general categories: (1) antioxidants such as ascorbic acid or citric acid that reduce oxidative stress and prevent oxidation of phenolic compounds [18,21]; (2) adsorbents such as activated charcoal or polyvinylpolypyrrolidone (PVPP) that bind phenolic compounds rendering them less toxic [21,23]. In addition, amino acids like glycine, asparagine, l-glutamine, and l-proline are also added to callus culture media as a source of reduced nitrogen that are readily metabolized by plant cells and stimulate faster cell growth and development [20]. The beneficial role of using l-glutamine to inactivate the oxidized products of polyphenols is also reported in tea [4] and Patchouli (*Pogostemon cablin*) [24]. In addition, use of acetosyringone has become a routine exercise in the *Agrobacterium*-mediated plant transformation for *Agrobacterium* *vir* gene induction [15,25]. Nevertheless, the effects of acetosyringone are highly dependent upon dose, specific explant type, species, genotype, and culture conditions. It may act as a bacteriostatic agent at higher concentrations [26], as a causative agent for necrotic reaction in rice calli [20], and lead to tissue browning and mortality of protocorm-like bodies (PLBs) of Orchid [27].

Although different chemicals (antioxidants/adsorbents) can be used in culture media to reduce oxidative browning, they may have a negative effect on *Agrobacterium* growth, *vir* gene expression, and subsequently on plant transformation. Studies on tea transformation improvement using different antioxidants or adsorbents are scarcely reported. *Agrobacterium rhizogenes* mediated generation of transgenic root (hairy root) could be an easy and efficient tool for the quick validation of a transgene, whereas the generation of transgenic tea plants is difficult and takes a long time. In addition, modulation of gene expression in transgenic hairy roots can be employed for studies on tea biology and biotechnology with an emphasis on tea roots and root–shoot interactions. Therefore, in this study, manipulation of the culture medium components with different antioxidants or adsorbents was conducted for significantly improved transgenic hairy root generation from the cotyledon derived calli of *C. sinensis*. Our study led to the establishment of a protocol for tea hairy root production with improved efficiency.

## 2. Results

### 2.1. Effect of Medium Supplements on Minimizing Callus Browning for Regular Subculture

**Figure 1:**
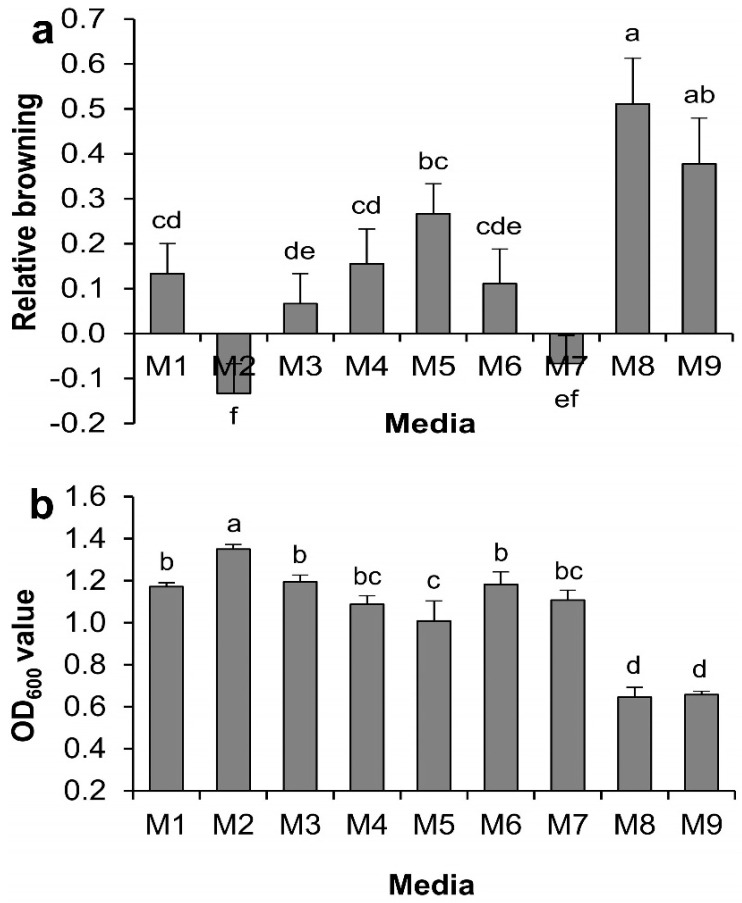
Effect of medium supplements on tea callus browning and *Agrobacterium* growth. (**a**) On tea callus browning in a regular subculture; (**b**) on *Agrobacterium* growth in Luria Bertani (LB) broth. Data show the mean ± standard deviation (SD). Statistical significance was analyzed using ANOVA. Means followed by the same letter are not significantly different (*p* > 0.05).

For regular sub-culturing, excision of explants is often required for proliferation. The effects of different tested supplements (M1–M9, Table 1) on tea callus browning were first examined for regular sub-culturing. Browning of tea calli grown on each medium was indexed 16 days after sub-culture plating relative to those examined immediately after callus plating on the same medium. A significant difference was found among the nine different supplements (Figure 1a). The lowest browning index occurred in M2 (−0.13 ± 0.07) and comparable to M7 (−0.07 ± 0.07), both were negative, suggesting that the browning was alleviated after sub-culturing on the two media. A significant difference existed between the M1 control and M2, suggesting that a reduced level of sucrose (5 g·L^−1^) in combination with l-glutamine helped to mitigate callus browning. Moreover, the relative browning value on M7 was also low and comparable to the lowest M2, suggesting that a combination of l-glutamine with citric acid at the tested concentrations also reduced callus browning. Relative browning values in the M3 and M6 was low but comparable to the M1, suggesting that supplementation of l-glutamine alone or combined with PVPP had some impact on browning minimization. Supplementation of acetosyringone in M4, and dithiothreitol (DTT) combined with others in M5, M8, and M9, increased callus browning and the increase was significant in M8, as well as M9 compared to the control. These results suggest that supplementation of acetosyringone and DTT at the tested concentrations had negative effects on controlling browning.

### 2.2. Effect of Medium Supplements on Agrobacterium Growth Grown in Luria Bertani (LB) Broth

The effect of different supplements on *Agrobacterium* growth was first examined in the Luria Bertani (LB) broth. Significant variations in *Agrobacterium* growth were observed due to different supplements after 7 h of culture (*p* < 0.01) (Figure 1b). The highest *Agrobacterium* growth was observed in the medium M2, followed by M3, M6, M1, M7, and M4. These data indicated that reduced level of sucrose favored *Agrobacterium* growth while l-glutamine and PVPP exerted no negative effect; citric acid and acetosyringone supplementation had little negative effect on *Agrobacterium* growth although no significant differences existed between the M3, M6, M7, M4, and the M1 control. On the other hand, *Agrobacterium* growth was significantly suppressed in the media M8 and M9, followed by in M5 compared to the control, suggesting that DTT supplementation at the tested concentration had an inhibitory effect on *Agrobacterium* growth.

### 2.3. Effect of Medium Supplements on vir Gene Expression

**Figure 2:**
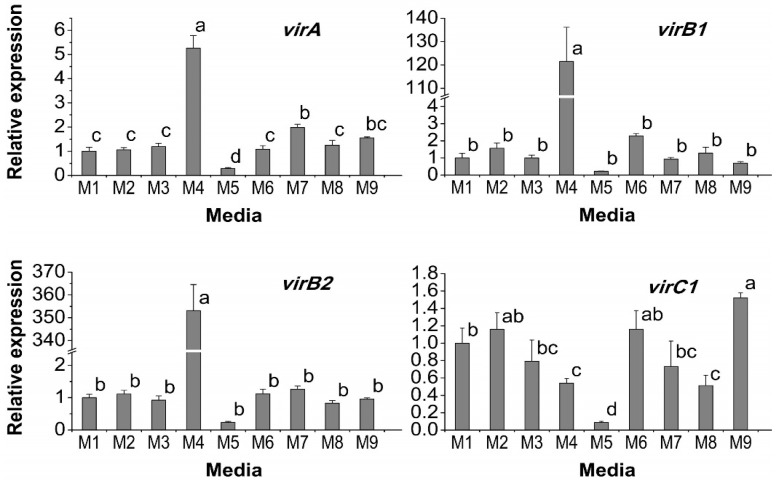

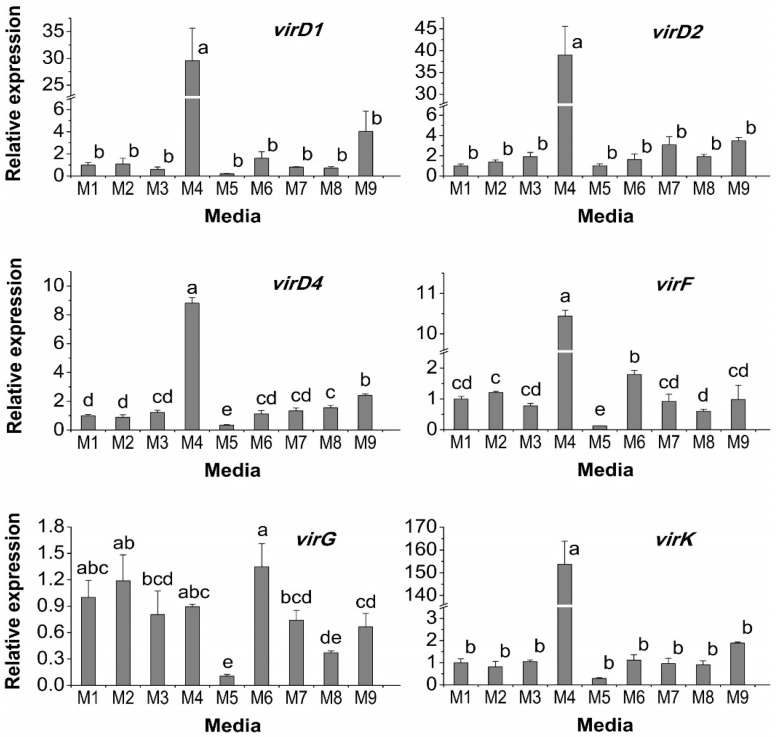
Effect of medium supplements on *Agrobacterium* *vir* gene expression after 7 h of culture in the LB broth. Transcript levels of *virA*, *virB1*, *virB2*, *virC1*, *virD1*, *virD2*, *virD4*, *virF*, *virG*, and *virK* genes were detected by quantitative reverse transcription PCR (qRT-PCR) and normalized to a chromosomal gene *Atu0972* and compared to the control (M1). Data show the mean ± SD. Statistical significance was analyzed using ANOVA. Means followed by the same letter are not significantly different (*p* > 0.05).

Expression of *vir* genes is essential for a successful *Agrobacterium*-mediated gene transfer. Acetosyringone is a well-known inducer of *vir* genes but other medium supplements may also affect the *vir* system; hence, this study was carried out to observe the effect of the supplements on *vir* induction. The expressions of all the tested *vir* genes were significantly affected by different medium supplements (Figure 2). Acetosyringone supplementation in the M4 medium significantly increased the expression of all of the *vir* genes in different extents compared to the control M1 except *virC1* and *virG*. Citric acid in combination with l-glutamine in M7 significantly enhanced *virA* expression. In addition, PVPP in combination with l-glutamine in M6 significantly enhanced the expression of *virF*. *virB1*, *virC1*, and *virG* were also more highly expressed, though not significantly (*p* > 0.05). Citric acid in combination with l-glutamine, DTT, and PVPP in the M9 enhanced the expression of *virC1* and *virD4*. It was observed that in the medium M5 supplemented with DTT and l-glutamine, expression of the *vir* genes were suppressed and in case of *virA*, *virC1*, *virD4*, *virF*, and *virG*, the suppression was highly significant compared with the control. The expression level of these genes in the M3 medium supplemented with l-glutamine suggests that the suppression was caused by the DTT but not by the l-glutamine supplementation. It was observed that the overall expression of all the *vir* genes was enhanced 72.29-fold in M4, 1.82-fold in M9, 1.43-fold in M6, and 1.28-fold in M7 compared to the control. These results suggest the beneficial role of PVPP and citric acid supplementation on the induction of *vir* genes, although not comparable with the potent inducer acetosyringone.

### 2.4. Effect of Medium Supplements on Agrobacterium Growth and Tissue Browning in the Inoculation Study

For *Agrobacterium*-mediated genetic transformation, virulent *Agrobacterium* infection and co-cultivation with explants are essential. *Agrobacterium* growth can be affected by explant-released chemical compounds, and browning of explant tissue can also be affected by *Agrobacterium* infection. Therefore, the effects of different medium supplements on *Agrobacterium* growth and callus browning were investigated in this study.

#### 2.4.1. Effects on *Agrobacterium* Growth

**Figure 3:**
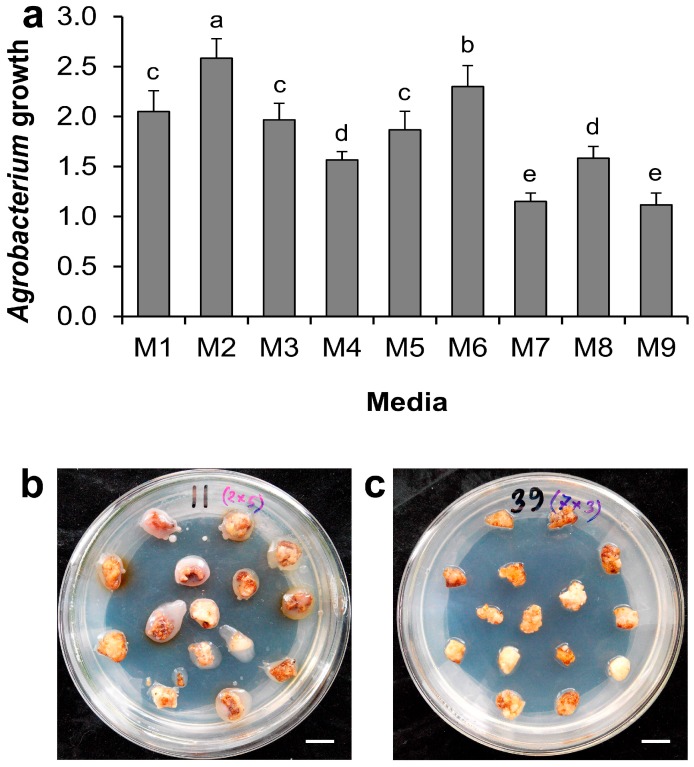
Effect of medium supplements on *Agrobacterium* growth during co-cultivation. (**a**) Effect of medium supplements on *Agrobacterium* growth. Data show the mean ± SD. Statistical significance was analyzed using ANOVA. Means followed by the same letter are not significantly different (*p* > 0.05); (**b**) plate showing the highest *Agrobacterium* growth in M2 medium; (**c**) plate showing the lowest *Agrobacterium* growth in M7 medium. Scale bar = 10 mm.

Significant impact of different supplements on *Agrobacterium* growth over the co-cultivation period was also noted in the inoculation study (Figure 3). *Agrobacterium* growth on medium M2 was significantly higher than that on M6, and both were higher than that on control M1. *Agrobacterium* growth on M3 and M5 was comparable with that on M1, suggesting that reduced sucrose or PVPP supplementation enhanced *Agrobacterium* growth, whereas l-glutamine or DTT supplementation had limited effects on *Agrobacterium* growth (Figure 3a). On the contrary, significantly suppressed *Agrobacterium* growth was found on media M4, M7, M8, and M9 compared with M1. Furthermore, no significant difference in *Agrobacterium* growth was noted between M4 and M8, or M7 and M9, indicating that suppression effect of collective supplementation of l-glutamine, DTT, and PVPP on *Agrobacterium* growth was comparable with acetosyringone. Meanwhile, supplementation with citric acid exhibited an even more pronounced negative effect on *Agrobacterium* growth than with acetosyringone since *Agrobacterium* growth was more pronounced on M4 than on M7 and M9. Figure 3b,c shows the *Agrobacterium* growth around inoculated calli on media M2 and M7, which displayed the highest and lowest *Agrobacterium* growth on them, respectively.

#### 2.4.2. Effects on Callus Browning

**Figure 4:**
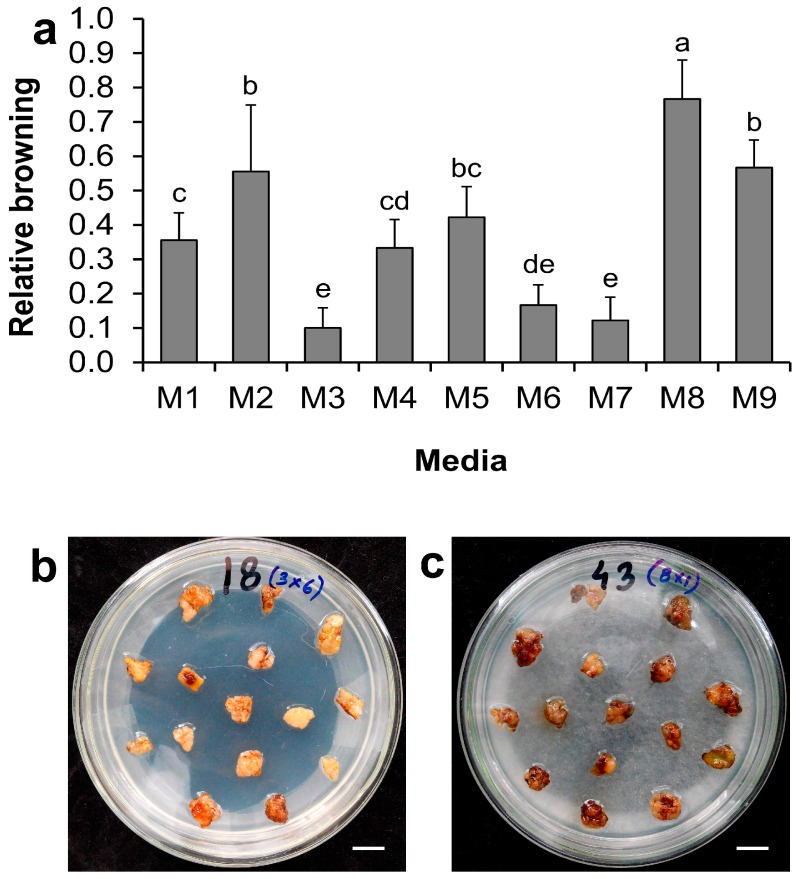
Effect of medium supplements on browning of tea calli after co-cultivation. (**a**) Effect of medium supplements on browning of tea calli. Data show the mean ± SD. Statistical significance was analyzed using ANOVA. Means followed by the same letter are not significantly different (*p* > 0.05); (**b**) plate showing the lowest browning in M3 medium; (**c**) plate showing the highest browning in M8 medium. Scale bar = 10 mm.

Freshly excised callus tissues were inoculated with *Agrobacterium* culture and co-cultivated with different medium supplements. Following co-cultivation, sub-culture was made for another 10 days on the corresponding medium with antibiotic (as described earlier) and then browning data were collected. Significantly lower relative browning occurred with the M3, M7, and M6 media compared with the M1 (Figure 4a). Browning was highest with the medium M8 (0.77 ± 0.12), followed by M9 (0.57 ± 0.09), M2 (0.56 ± 0.21), and M5 (0.42 ± 0.10), although the differences between the latter three were statistically insignificant. These results suggest that co-cultivation and post co-cultivation media supplemented with l-glutamine (0.1 g·L^−1^) and a standard sucrose level (30 g·L^−1^) resulted in 71.9% reduction of browning compared with that of the control. The media supplemented with l-glutamine along with citric acid or PVPP were also helpful in reducing browning (by 65.6% and 53.1%, respectively, compared with the control). In contrast, other antioxidants and adsorbents used either alone or in combination under the present study conditions were unable to reduce browning; even combinations of supplements in M8 and M9 enhanced browning significantly compared to the M1 control. In the case of the M2 medium, although this medium was supplemented with l-glutamine, use of a reduced dose of sucrose (5 g·L^−1^) enhanced browning significantly (1.6-fold higher) than the control. Figure 4b,c show the calli in media M3 and M8, which displayed the lowest and highest browning, respectively.

### 2.5. Effect of Medium Supplements on the Generation of Hairy Roots

It was observed from the inoculation experiment that *Agrobacterium* growth was better maintained in media M1, M2, M3, M5, and M6, whereas browning was minimum in media M3, M6, and M7. On the other hand, the highest browning was observed in M8, where *Agrobacterium* growth was also suppressed compared to the control. The medium M2 showed high *Agrobacterium* growth and callus browning. Thus, media M3 and M6, where both the parameters were better maintained, and media M2 and M8, where both the parameters were not desired, were selected for the transformation study to observe the impact of these two parameters on transformation. In addition, considering the *vir* gene induction effect, *Agrobacterium* cultures were treated with acetosyringone (150 μM) before the inoculation of calli, instead of being supplemented in the co-cultivation medium.

**Figure 5:**
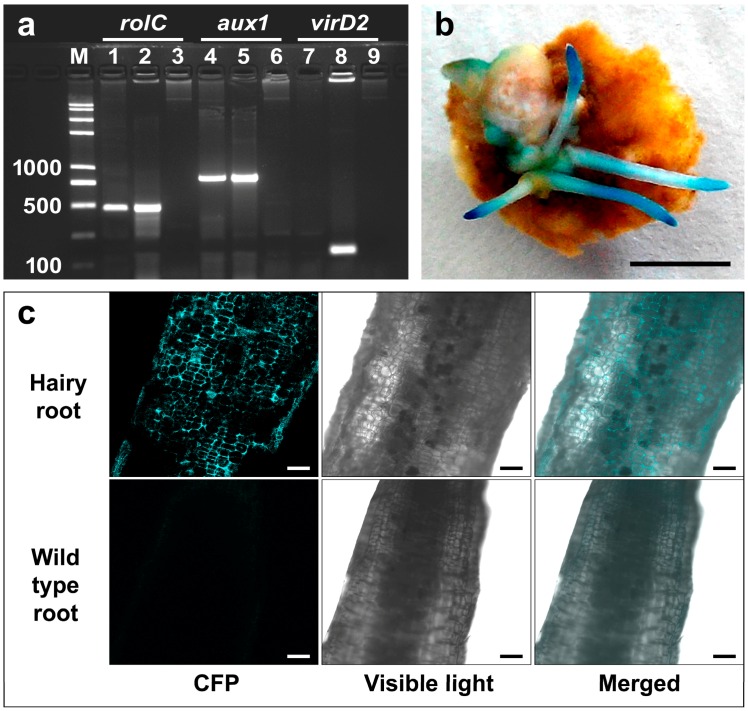
PCR amplification of the transgenes, and expression of either β-glucuronidase (GUS) or cyan fluorescent protein (CFP) in the tea hairy roots. (**a**) PCR confirmation of the transgenic nature of the hairy roots by the presence of transferred *Agrobacterium* genes *rolC* and *aux1*, and the absence of non-transferred *Agrobacterium* gene *virD*2 in the hairy roots; lane M, DNA marker; lanes 1, 4, and 7, DNA from hairy root; lanes 2, 5, and 8, DNA from bacteria (positive control); lanes 3, 6, and 9, DNA from wild-type roots (negative control). Expected sizes for the amplified fragments of the *rolC*, *aux1* and *virD2* are 486, 815, and 145 base pairs, respectively; (**b**) stable GUS expression in the hairy roots, scale bar = 5 mm; (**c**) fluorescent imaging of the hairy root and wild-type root for stable CFP expression, scale bar = 200 μm.

In this study, 64 hairy root lines were obtained from the inoculated tea callus explants. The callus explants without *Agrobacterium* infection did not produce any roots unless auxin was added to the culture medium. The initial appearance of hairy roots was observed around 31 days following inoculation on medium without auxin supplementation. In the hairy roots, transgenes *rolC* and *aux1* were PCR detected whereas *Agrobacterium* non-transgene *virD2* was not found, confirming that the detection of the two transgenes was resulted from successful gene transformation, rather than *Agrobacterium* contamination (Figure 5a). Histochemical assay for stable β-glucuronidase (GUS) expression or fluorescent imaging for stable cyan fluorescent protein (CFP) expression further validated the stable integration of a foreign gene in the transgenic hairy roots (Figure 5b,c), at an overall efficiency of 28% in the generated hairy roots.

**Figure 6:**
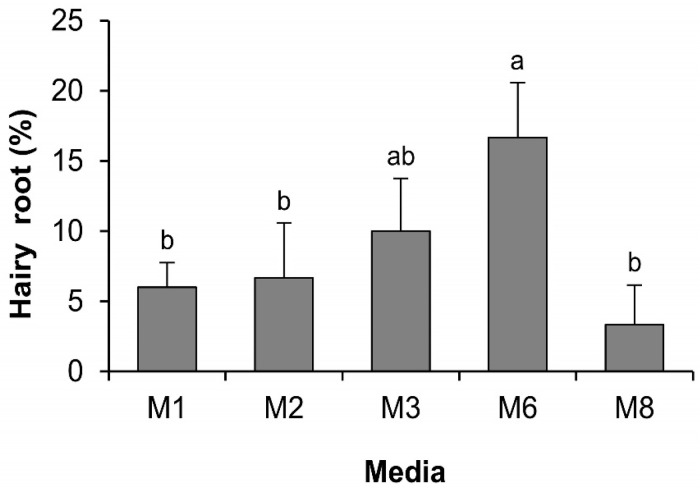
Effect of medium supplements on hairy root generation efficiency from tea calli. Data show the mean ± SD. Statistical significance was analyzed using ANOVA. Means followed by the same letter are not significantly different (*p* > 0.05).

Hairy root generation efficiencies varied significantly due to different medium supplements (Figure 6). The highest efficiency (16.7% ± 4.2%) was obtained with the M6 medium, followed by M3 (10.0% ± 4.0%), M2 (6.7% ± 4.2%), and M1 (6.0% ± 2.0%), and the lowest was obtained in the M8 (3.3% ± 3.1%). Comparing the hairy root generation efficiencies in the media M1, M2, and M3, it can be assumed that l-glutamine supplementation had positive effects and reduced level of sucrose had negative effects on hairy root generation. Moreover, supplementation with 0.1 g·L^−1^ l-glutamine in M3 resulted in 1.7-fold higher hairy root generation efficiency compared to M1. When the same amount of l-glutamine was supplemented with a phenolic adsorbent PVPP (5 g·L^−1^) in the M6 medium, the efficiency reached 2.8 times higher than the control. Further addition of 0.15 g·L^−1^ DTT with l-glutamine and PVPP in the M8 medium led to a dramatic decrease in the hairy root generation efficiency compared to M6, even lower than the control, suggesting the negative effects of DTT at the tested concentration on the generation of hairy roots from tea calli.

## 3. Discussion

A few transformation events of tea plants [13,14,28,29] and hairy root generation [30] have been reported in Assam tea varieties (*Camellia sinensis* var. *assamica*). The transformation study in Chinese tea varieties (*C. sinensis* var. *sinensis*) was scarcely reported compared to Assam varieties, probably due to genotypic variances in competency to transformation as observed in several studies [4,28]. Jeyaramraja and Meenakshi [28] tested five genotypes of Assam tea varieties and reported only one genotype (UPASI-3) was susceptible to *A. tumefaciens* strain LBA4404-mediated transformation. In another study with five other genotypes of Assam tea, Kumar et al. [4] found the highest transient GUS expression in the genotype TV1 using *A. tumefaciens* strain EHA105. These findings indicated that in addition to genotype, virulent *Agrobacterium* strain can also be a determinant of transformation success in tea. Later, in the previous study [5] we found that among different *Agrobacterium* strains, only *A. rhizogenes* strain ATCC15834 was capable of inducing hairy root from all explants of the Chinese tea genotype “Nong Kangzao.” This result suggested that transformation competence of a tea genotype was also largely specific to a particular *Agrobacterium* strain. Among the other related factors, bactericidal effects of tea polyphenols and explant tissue browning during the *Agrobacterium*-mediated transformation process were reported as the major bottleneck in tea transformation [3,4]. Therefore, in the present study an attempt has been made to overcome these problems through manipulating the culture medium components, largely using different antioxidants and adsorbents. In addition, observation was performed for its impact on the transformation efficiency by generating hairy roots from the calli of “Nong Kangzao” seedlings using *A. rhizogenes* strain ATCC15834.

Investigations on the tested supplements indicated that, selection of an appropriate antioxidants/adsorbents or their mixtures is extremely important for controlling callus tissue browning, because some combinations not only failed to control browning, but also increased browning significantly than the control. Further investigation showed that tea callus browning was affected by medium components and by *Agrobacterium* infection as well. *Agrobacterium* involvement overall resulted in a greater than two-fold increase in the relative browning values in tea calli. A reduced level of sucrose or supplementation of citric acid resulted in the lowest values of relative callus browning when *Agrobacterium* was not applied. Once calli were inoculated with *Agrobacterium*, a reduction in sucrose led to the highest level of *Agrobacterium* growth and extensive callus browning, while citric acid supplementation along with l-glutamine resulted in suppressed *Agrobacterium* growth and minimum callus browning. A similar result was obtained with citric acid supplementation by Jartoodeh et al. [31], who noted that the highest survival percentage of pear explants is obtained with addition of citric acid in the culture media. *Agrobacterium* growth suppression by citric acid was also reported by Daly [32] due to its antibacterial activity. Supplementation of DTT combined with others increased callus browning relative to the control, suggesting the toxic effect of DTT at the tested concentration. This is in agreement with the study by Cheng [33], who reported that the antioxidant itself may cause cell death and reduce the survival rate of target plant cells. A similar trend in *Agrobacterium* growth was observed both in the LB broth and during co-cultivation due to the different supplements; the only important change noticed was that in the case of LB broth the growth of *Agrobacterium* in M6 was comparable to that in M1, whereas during co-cultivation, the M6 medium showed significantly higher *Agrobacterium* growth than the control M1. These findings suggest some bactericidal effects of calli-released phenolics existed in M1, and the same phenolics were stabilized by l-glutamine and PVPP at the tested concentrations in M6. Kumar et al. [4] reported that amino acids like l-glutamine can react with quinones to form adducts via the Schiff’s base I and II, and finally, amino catechins, and this in turn inactivates the quinones or the oxidized products of polyphenols. On the other hand, PVPP, a strong adsorption agent, can effectively adsorb polyphenol from the media and might not exert harmful effects on the medium components due to its water-insoluble property [34,35]. Thus, these beneficial properties of l-glutamine and PVPP at the tested concentrations in M6 inhibited the activity of polyphenols and/or quinones to some extent so that tea callus browning was suppressed and *Agrobacterium* growth was well maintained compared to other tested media.

Induction of *Agrobacterium* *vir* genes and their subsequent actions are essential for transformation [17]. The mechanism of *Agrobacterium* *vir* gene induction and their subsequent actions on transformation have been extensively studied [36,37]. In brief, *virA* recognizes the plant signal molecules and *virG* is the response regulator which activates all genes in the regulon; *virC1*, *virD1*, *virD2*, and *virE2* are involved in transfer DNA (T-DNA) complex processing; *virB* and *virD4* constitute the type IV protein secretion system for T-DNA translocation [37,38]. In the present study, we investigated the transcript level of different *vir* genes to observe whether any of the medium supplements would exert notable positive/negative effects on the *vir* induction. Our result showed that overall *Agrobacterium* *vir* system was strongly induced by acetosyringone supplementation, followed by a weak induction (compared to acetosyringone) in the M9 medium (supplemented with l-glutamine and PVPP with others) and then in the M6 medium (supplemented with l-glutamine and PVPP). Supplementation of DTT along with l-glutamine suppressed all the *vir* genes. These results indicated that DTT supplementation had negative effect on the *vir* system. Reports are very few to recognize the compounds of which supplementation negatively affects the *Agrobacterium* *vir* system. In an earlier study, Anand et al. [39] identified negative effects of salicylic acid on the *vir* induction system and here we identified the DTT as an inhibitor of the *vir* system. Our findings also indicated that *vir* induction was independent of *Agrobacterium* growth. However, the overall results obtained in this simulation experiment might not show the real situation of the *vir* gene induction in the transformation experiments because the phenolic signal molecules released from the callus explants would also act on the *Agrobacterium* *vir* genes.

In the transformation study we found that, among the five selected media (M1, M2, M3, M6, and M8), the M6 medium supplemented with l-glutamine and PVPP generated hairy roots at the highest efficiency. Comparing the *Agrobacterium* growth and browning data in these media, it was observed that the M6 medium showed significantly higher *Agrobacterium* growth and lower tissue browning with exception to M2 and M3, respectively. Though the *Agrobacterium* growth was higher in M2 but it showed about 3.3 times higher browning than M6; in the case of M3, although browning was comparable, the *Agrobacterium* growth was significantly lower than M6. Medium M8 showed the highest browning and lowest *Agrobacterium* growth. These results suggested that adequate *Agrobacterium* growth and minimized explant browning favored tea callus transformation. In addition, M6 medium induced the expression of *virG,* as well as *virF* even though *virF* was more significantly induced by acetosyringone in M4. Moreover, expression level of *virC1* in M6 was high, although not as high as in M9. From the available literature, we found that *virA* and *virG* together code a two-component (VirA–VirG) system and activate the transcription of the other *vir* genes [36,40]. In addition, *virF* confers substrate specificity to the SCF (SKP1-CUL1-F-box protein) proteasomal machinery that is responsible for the releasing the T-DNA from the T-complex and helps to direct the single stranded T-DNA (ssT-DNA) to the nucleus within plant cell [41]. Some reports also suggested that *virC1* [42] and *virF* [43] were involved in determination of the host range. Huffman [44] reported that with other *vir* genes, *virG* contribute to the “hypervirulence” of particular strains. Again, a significant increase in the expression of most of the *vir* genes by the tea callus extract was also reported by Song et al. [5]. Thus it can be speculated that with the above induction effect of the medium components in M6, callus-released phenolic signal molecules also acted synergistically to induce the *vir* system as effectively as or more so than the others. Putting all these results together, it can be said that adequate *Agrobacterium* growth with high virulence and minimized explant browning in the M6 medium, supplemented with l-glutamine and PVPP at the tested concentrations, was responsible for the generation of hairy roots at highest level. On the contrary, the lowest hairy root generation efficiency in the M8 medium was also in accordance with the highest tissue browning with lowest *Agrobacterium* growth and *vir* gene expression in this medium.

The present study showed that amendment of culture medium components can significantly affect the *Agrobacterium* growth, *vir* gene expression, and explant tissue browning, and subsequently showed its effect on hairy root generation efficiency. In this study, we have achieved up to 16.7% hairy root generation efficiency from the cotyledon-derived calli of Chinese tea variety “Nong Kangzao” by amending the culture medium conditions. Additionally, the reporter gene *gusA* or *cfp* was also stably expressed in the transgenic hairy roots. These findings could probably open up a new door to work with the specific genes of interest to manipulate their expressions *in planta* with the Chinese tea varieties. Further studies are required for a better understanding of the mechanisms controlling the transgenic hairy root generation from the callus tissue, and the mechanisms could be an important area of further research.

## 4. Materials and Methods

### 4.1. Explant Preparation and Callus Induction

**Figure 7:**
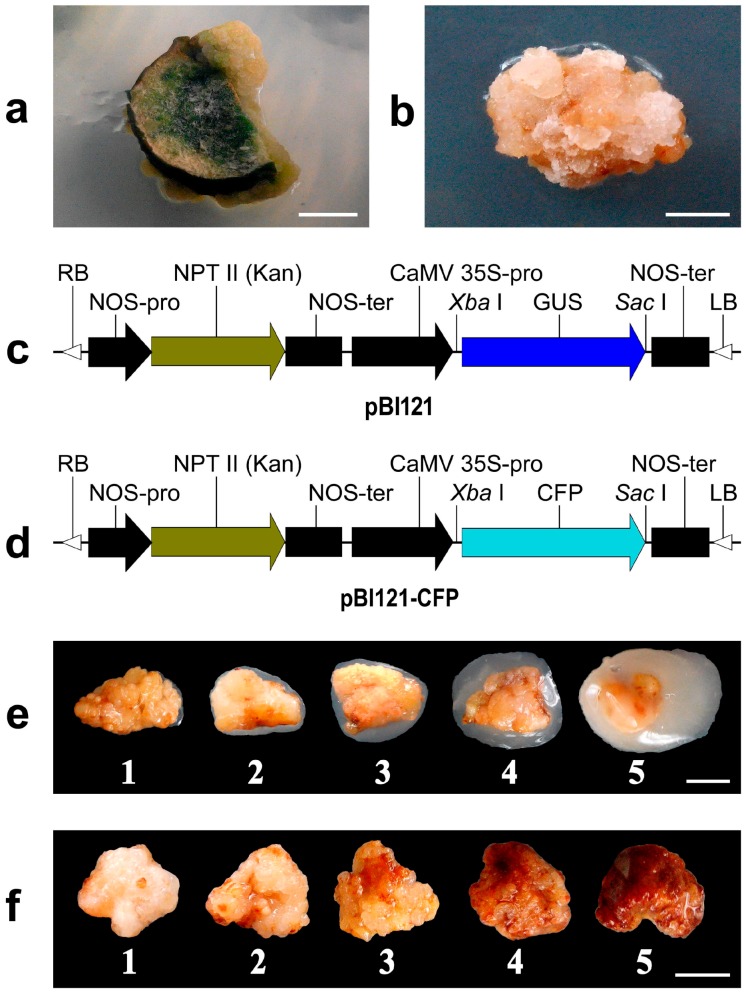
Plant materials and plant expression vectors used in this study. (**a**) Callus developed on the tea cotyledon explant; (**b**) callus used for sub-culturing and inoculation; (**c**) schematic diagram of the vector pBI121; (**d**) schematic diagram of the modified vector pBI121-CFP; (**e**) index for measuring *Agrobacterium* growth; (**f**) index for measuring extent of browning. Scale bar = 5 mm. RB, Right boarder; LB, Left boarder; NPT, Neomycin phosphotransferase; NOS, Nopaline synthase; GUS, β-glucuronidase; CFP, cyan fluorescent protein.

Mature seeds were collected from a seven-year-old tea plant (*Camellia sinensis* var. *sinensis*) cv. “Nong Kangzao” grown at the experimental tea farm of Anhui Agricultural University, Hefei, China. The seeds with shells (seed coats) were surface disinfected with 70% (*v*/*v*) ethanol for 2 min and then with 1.5% (*v*/*v*) sodium hypochlorite for 10 min, followed by three washes with sterile distilled water. Surface sterilized seeds were blot-dried and de-shelled under aseptic condition. For germination, the seeds were then placed on half-strength Murashige and Skoog (MS) basal salts medium [45] supplemented with 30 g·L^−1^ sucrose and 7 g·L^−1^ agar (pH 5.7) and maintained at 25 ± 1 °C with a 16 h photoperiod. For callus induction, green cotyledons were excised from three-week-old seedlings, cut into 4–5 mm slices and then placed into Petri plates containing half-strength MS basal salts medium [45] supplemented with 30 g·L^−1^ sucrose, 2 mg·L^−1^ 2,4-dichlorophenoxyacetic acid (2,4-D), 0.2 mg·L^−1^ benzyl adenine (BA), and 7 g·L^−1^ agar (pH 5.6). The cotyledon cultures were incubated at 25 ± 1 °C with a 16 h photoperiod. After two sub-cultures at a four-week interval, the generated calli (Figure 7a) were separated from the cotyledons and sub-cultured as different lines onto the same callus induction medium. After four weeks, one vigorously growing line was selected and sub-cultured two times in the dark at four weeks interval in the same medium. The calli (Figure 7b) were then continuously sub-cultured every four weeks on a Gamborg (B5) medium [46] with low hormone levels (0.25 mg·L^−1^ 2,4-D and 0.1 mg·L^−1^ Kinetin) with pH 5.5 at 25 ± 1 °C in the dark. Actively growing yellowish friable calli were selected for this study.

### 4.2. Agrobacterium Strain and Transformation Vector

*Agrobacterium rhizogenes* strain ATCC 15834 and the binary vector pBI121 (Figure 7c) were used in this study. In addition, for non-damage observation of the expressed transgene *cfp*, encoding for cyan fluorescent protein (CFP) obtained from pAmCyan Vector (Clontech, Cat. No. 632440, Beijing, China), the binary vector pBI121-CFP was constructed (Figure 7d) by replacing the *gusA* gene at the *Xba* I/*Sac* I sites of pBI121 with *cfp*. Thus the normal vector pBI121 contained *gusA* and *npt*II, and the modified vector pBI121-CFP contained *cfp* and *npt*II as reporter and selective genes, respectively. The two vectors were introduced separately into the *Agrobacterium* through electroporation and these two transformed *Agrobacterium* lines were preserved at −80 °C for further use.

### 4.3. Medium Supplements

In the present study, to achieve optimum *Agrobacterium* growth, *vir* gene expression and reduced calli browning, nine different media (M1–M9) were prepared with different supplements using l-glutamine (Solarbio, Beijing, China), acetosyringone (Solarbio, China), DTT (Sangon Biotech, Shanghai, China), PVPP (Solarbio, China), citric acid (Sinopharm Chemical Reagent Co., Ltd., Shanghai, China), and sucrose (Xilong Chemical Co., Ltd., Shantou, China). In addition, 2.0 mg·L^−1^ indole-3-butyric acid (IBA) and 0.2 mg·L^−1^ BA (both from Biosharp, Hefei, China), and 1 mL·L^−1^ Gamborg’s vitamin solution 1000× (Sigma, Saint Louis, MO, USA) were added to the media listed in Table 1. The different types of media used in this study were solidified with 7 g·L^−1^ agar and the pH was adjusted to 5.6 before autoclaving at 121 °C for 20 min. l-Glutamine, acetosyringone, DTT, citric acid, hormones, and antibiotics were filter-sterilized and added when the autoclaved media was cooled to about 50 °C.

### 4.4. Experimental Design

To analyze the effect of medium supplements (M1–M9) on *Agrobacterium* growth, *vir* gene expression, browning of calli, and hairy root generation, four different experiments were conducted: (i) Tissue culture study: the calli were grown on MS media (M1–M9) to observe the efficiency of the supplements to inhibit browning; (ii) *Agrobacterium* growth and *vir* gene assay: *Agrobacterium* cultures were grown in LB broth with M1-M9 supplements to observe the effect on *Agrobacterium* growth and *vir* gene expression; (iii) Inoculation study: *Agrobacterium* cultures were pre-treated with acetosyringone and used for co-cultivation to determine the efficiency of M1–M9 supplements on *Agrobacterium* growth and prevention of calli browning; and (iv) Transformation study: similar to inoculation study, but the experiment was conducted with a few selected supplements (M1, M2, M3, M6, and M8) favorable or unfavorable for callus browning prevention and adequate *Agrobacterium* growth, to observe the efficiency of the selected supplements on hairy root generation. All of the experiments were conducted in a completely randomized design with three to six replications detailed in Table A1. For the first and third experiment, each Petri plate containing 15 explants was considered as a single replication and the fourth experiment was repeated thrice with 50 explants per treatment.

### 4.5. Agrobacterium Growth and vir Gene Expression Assay Using LB Broth

For the *Agrobacterium* growth and *vir* gene expression assay, LB broth were prepared with nine different supplements (M1–M9, as listed in Table 1 with antibiotic rifampicin and kanamycin both at 50 mg·L^−1^ but without MS salts). The overnight culture of *Agrobacterium* strain ATCC 15834 harboring pBI121 was prepared and equal aliquots were centrifuged at 2600× *g* for 5 min. *Agrobacterium* pellets were re-suspended with each 100 mL LB broth and the initial OD_600_ values were adjusted to 0.15. The cultures were then incubated at 28 °C in the dark on a rotary shaker (180 rpm). The change in OD_600_ values were recorded after 7 h of incubation. As PVPP was insoluble in the media, the OD_600_ values of the cultures were measured after pelletizing PVPP by centrifugation at 150× *g* for 5 min. The corresponding media without *Agrobacterium* were used as references for measuring the OD_600_ of the inoculated cultures. *Agrobacterium* cells were then harvested and total RNA was isolated using an RNAprep pure Cell/Bacteria Kit (Tiangen Biotech., Beijing, China) according to the manufacturer’s instructions. The RNA was reverse-transcribed using a PrimeScript™ RT reagent Kit with gDNA Eraser (TaKaRa, Dalian, China). Real time quantitative polymerase chain reaction (qPCR) was performed using the top Green qPCR SuperMix Kit (TransGen, China) with gene specific primers for *virA*, *virB1*, *virB2*, *virC1*, *virD1*, *virD2*, *virD4*, *virF*, *virG*, and *virK* (Table S1). *Atu0972*, an *Agrobacterium* chromosomal gene, was used as a reference gene according to Anand et al. [47]. qPCR reactions were performed on a Bio-Rad CFX96 platform and programmed as follows: 3 min at 95 °C, 40 cycles of 10 s at 95 °C, 40 s at 55 °C; followed by a 5 s melting curve analysis of 65–95 °C, in 0.5 °C increments. Transcript data were analyzed using 2^−∆∆*C*t^ method [48] with the CFX Manager™ software (Bio-Rad Laboratories, Inc., Hercules, CA, USA). All measurements were conducted in triplicate.

### 4.6. Agrobacterium Inoculation to Callus and Induction of Hairy Roots

For the induction of hairy roots in tea calli, overnight culture of the transformed *A. rhizogenes* grown in LB broth media containing antibiotic rifampicin and kanamycin (both at 50 mg·L^−1^) was obtained and an aliquot of the *Agrobacterium* suspension was transferred to fresh LB medium containing the same antibiotics for a large scale of *Agrobacterium* culture preparation with OD_600_ value around 0.8. *Agrobacterium* cells were pelleted by centrifugation at 2100× *g* for 10 min and the pellet was re-suspended in equal volume of MS liquid medium supplemented with 150 μM acetosyringone. The culture was incubated for another 30 min to induce the *vir* genes and the final OD_600_ value was adjusted to 0.8 before inoculation. *Agrobacterium* cultures were prepared separately for both the transformed *Agrobacterium* lines (one harboring pBI121 and another pBI121-CFP) and were mixed together before inoculation of explants. Tea callus pieces (8–10 mm) were submerged in the mixed *Agrobacterium* culture for 20 min, blotted on sterile filter paper to remove excess *Agrobacterium*, and then plated to different types of media (Table 1) for co-cultivation (20 °C in the dark for six days). Following co-cultivation, the callus pieces were rinsed four times with sterile distilled water to remove *Agrobacterium*, then subjected to one additional rinses with MS liquid containing 800 mg·L^−1^ carbenicillin, blotted on filter paper, and plated to the corresponding fresh medium supplemented with antibiotic carbenicillin (500 mg·L^−1^). The cultures were incubated at 25 ± 1 °C in the dark for another 10 days. After that they were continuously sub-cultured every three weeks into the hormone-free MS medium supplemented with 30 g·L^−1^ sucrose, 1 mg·L^−1^ l-glutamine, and 500 mg·L^−1^ carbenicillin at 25 ± 1 °C in the dark for the generation of hairy roots. In the third experiment, all procedures were the same except that the calli were maintained for just 10 days following co-cultivation to collect the browning data. In the first experiment the calli were cultured without *Agrobacterium* inoculation.

### 4.7. Data Collection

Indexing of *Agrobacterium* growth and explant browning in the inoculation study was conducted by direct visual observation combined with high resolution pictures, which were taken at three different time points of the experiment: T_1_, immediately following inoculation of calli; T_2_, on the sixth day of co-cultivation; T_3_, 10 days after the co-cultivation. Pictures taken at T_1_ and T_3_ were used to index browning within this period and the pictures taken at T_2_ were used to measure *Agrobacterium* growth. Based on visual observation and all the pictures taken, two different series of indices were generated, one for *Agrobacterium* growth (Figure 7e) and another for the extent of browning (Figure 7f). By comparing explants with the index scores (Little or no = 1, below average = 2, average = 3, above average = 4, and high = 5), each explant was individually assigned a score for both browning and *Agrobacterium* growth. The mean data from all the explants within a plate represented data for one replication. In this way the data from replications of all the treatments were collected. To measure the extent of browning, the mean browning data collected at time T_1_ from one replication was deducted the mean of its T_3_ data, and was recorded as relative browning (compared to T_1_). Browning data of the tissue culture study was collected in a similar way. Data were also collected after generation of hairy roots and the hairy root generation efficiency was calculated as the percentage of explants producing hairy root to the total number of explants inoculated.

### 4.8. Analysis of the Hairy Roots Using PCR

To verify the transgenic nature of the putative hairy roots, PCR analyses were performed according to Medina-Bolivar et al. [49]. In brief, using the gene specific primers, transferred *Agrobacterium* genes *rolC* and *aux1* were expected to be amplified from the hairy roots only. Meanwhile, PCR detection of the non-transferred *Agrobacterium* gene *virD*2 was performed to examine *Agrobacterium* contamination. Genomic DNA samples were extracted from both the putative hairy roots and wild-type roots using the MiniBEST Plant Genomic DNA Extraction Kit (TaKaRa, Dalian, China). The following primer sequences *rolC*, 5′-TGTGACAAGCAGCGATGAGC-3′ and 5′-GATTGCAAACTTGCACTCGC-3′; *aux1*, 5′-CCAAGCTTGTCAGAAAACTTCAGGG-3′ and 5′-CCGGATCCAATACCCAGCGCTTT-3′; *virD2*, 5′-GAGTGGGCAGCCGAGATGTT-3′ and 5′-ATCTTCAGCCAGCCGTGTCC-3′ were used to amplify the fragment length of 487, 815 and 145-bps, respectively. PCR was performed in a 25 μL reaction volume containing 1 Unit of Easy-Taq DNA Polymerase (TransGene, Beijing, China), 10 ng genomic DNA, 1 μL of each primer (10 mmol·L^−1^), 2.5 μL 10× buffer, 2 μL dNTP (2.5 mmol·L^−1^), and 17.5 μL water. Amplification was performed in a programmable Thermal cycler (Bio-Rad S1000) as follows: initial denaturation at 95 °C for 3 min, 30 cycles of amplification (95 °C for 30 s, 58 °C for 30 s and 72 °C for 1 min, and 72 °C for 10 min). Five microliters of each PCR product was resolved on 1.5% agarose gel and stained with ethidium bromide at 0.5 μg·mL^−1^ for visualization of the bands. At least two replicates were carried out for each PCR analysis.

### 4.9. β-Glucuronidase (GUS) and Cyan Fluorescent Protein (CFP) Expression in the Hairy Roots

The histochemical assay for *gusA* gene expression was performed as previously described by Jefferson et al. [50]. In brief, the hairy roots with or without callus explants were submerged into GUS staining solution (2 mM X-Gluc, 500 mM phosphate buffer, 10 mM EDTA, and Triton x-100) for 12 h incubation at 37 °C. The explants were soaked in 70% ethanol and examined for blue color development. CFP expression of the hairy roots was detected by confocal laser scanning microscope (Olympus FV1000, Tokyo, Japan).

### 4.10. Statistical Analysis

All the experiments were carried out at least with independent biological duplicates. The results were expressed as mean value ± standard deviation (SD). Data were analyzed using ANOVA, and statistical differences between means were compared by Duncan’s multiple range test (DMRT), using the statistical package MSTAT [51].

## 5. Conclusions

This study demonstrated significant impact of medium supplements on *Agrobacterium* growth, *vir* gene expression and browning of explants, and subsequently on the generation efficiency of hairy roots from tea calli. Citric acid supplementation (0.1 g·L^−1^) suppressed tea callus browning and *Agrobacterium* growth, whereas a reduced sucrose level (5 g·L^−1^) enhanced callus browning and *Agrobacterium* growth upon application of *Agrobacterium* infection and co-culture. In contrast, a reduction in sucrose suppressed browning when *Agrobacterium* was not applied. The medium supplemented with l-glutamine and PVPP was able to control tea callus browning and maintain *Agrobacterium* growth over the co-culture period with enhanced virulence. Thus, pretreating *Agrobacterium* with acetosyringone (150 μM) prior to callus inoculation and supplementing co-cultivation media with l-glutamine (0.1 g·L^−1^) and PVPP (5 g·L^−1^) led to a high level of hairy root generation efficiency from the cotyledon derived calli of Chinese tea variety “Nong Kangzao.”

## Figures and Tables

**Table 1:** List of media used in this study.

Medium	Supplements
M1	MS salts + Sucrose (30 g·L^−1^) (control)
M2	MS salts + Sucrose (5 g·L^−1^) + l-glutamine (0.1 g·L^−1^)
M3	MS salts + Sucrose (30 g·L^−1^) + l-glutamine (0.1 g·L^−1^)
M4	MS salts + Sucrose (30 g·L^−1^) + acetosyringone (150 μM)
M5	MS salts + Sucrose (30 g·L^−1^) + l-glutamine (0.1 g·L^−1^) + DTT (0.15 g·L^−1^)
M6	MS salts + Sucrose (30 g·L^−1^) + l-glutamine (0.1 g·L^−1^) + PVPP (5 g·L^−1^)
M7	MS salts + Sucrose (30 g·L^−1^) + l-glutamine (0.1 g·L^−1^) + Citric Acid (0.1 g·L^−1^)
M8	MS salts + Sucrose (30 g·L^−1^) + l-glutamine (0.1 g·L^−1^) + DTT (0.15 g·L^−1^) + PVPP (5 g·L^−1^)
M9	MS salts + Sucrose (30 g·L^−1^) + l-glutamine (0.1 g·L^−1^) + DTT (0.15 g·L^−1^) + PVPP (5 g·L^−1^) + Citric Acid (0.1 g·L^−1^)

MS, Murashige and Skoog; DTT, dithiothreitol; PVPP, polyvinylpolypyrrolidone.

## Supplementary Materials

Supplementary materials can be found at http://www.mdpi.com/1422-0067/17/7/1132/s1.

## Appendix A

**Table A1:** Design of the experiments in detail.

Experimental Parameters	Description
**First experiment (Tissue culture study)**	Completely randomized design
Treatments: MS media with different supplements	9 (M1, M2, M3, M4, M5, M6, M7, M8, and M9)
Replications	3
Total experimental units	27 (9 × 3 × 15 explants)
**Second experiment (*Agrobacterium* growth and *vir* gene assay**)	Completely randomized design
Treatments: LB broth media with different supplements	9 (M1, M2, M3, M4, M5, M6, M7, M8, and M9)
Replications	3
Total experimental units	27 (9 × 3)
**Third experiment (Inoculation study)**	Completely randomized design
Treatments: MS media with different supplements	9 (M1, M2, M3, M4, M5, M6, M7, M8, and M9)
Replications	6
Total experimental units	54 (9 × 6 × 15 explants)
**Fourth experiment (Transformation study)**	Completely randomized design
Treatments: MS media with different supplements	5 (M1, M2, M3, M6, and M8)
Replications	3 (50 explants in each)
Total experimental units	15 (5 × 3 × 50 explants)
