# Supplementary material for: Effect of Medium Supplements on Agrobacterium rhizogenes Mediated Hairy Root Induction from the Callus Tissues of Camellia sinensis var. sinensis
Source: Int J Mol Sci. 2016 Jul 15;17(7):1132. doi: 10.3390/ijms17071132 (PMC4964505; doi:10.3390/ijms17071132)
Supplement: Supplementary file 1 [file ijms-17-01132-s001.pdf]

Supplementary Materials: Effect of Medium Supplements on *Agrobacterium rhizogenes* Mediated Hairy Root Induction from the Callus Tissues of *Camellia sinensis* var. *sinensis*

Mohammad M. Rana, Zhuo-Xiao Han, Da-Peng Song, Guo-Feng Liu, Da-Xiang Li, Xiao-Chun Wan, Alagarsamy Karthikeyan and Shu Wei

Table S1. *Agrobacterium* virulence (*vir*) genes and primers used for gene transcript quantification.

Gene	Accession No.	Primer	Primer Sequence
virA	GCA_000705135.2	virA_F	5′-TCCTCGAAGAAGCCCGTCAG-3′
		virA_R	5′-CCAGAGCGGCGATCTTGTCT-3′
virB1	GCA_000705135.2	virB1_F	5′-CACTGGCAGAACCAGGCTCA-3′
		virB1_R	5′-AGGGCTCCGTCAGGTGTCAG-3′
virB2	GCA_000705135.2	virB2_F	5′-AATCGCCTCTCGCTCTCGAA-3′
		virB2_R	5′-CATTGTGGCTGGGTCAGTGC-3′
virC	GCA_000705135.2	virC_F	5′-TGCAAGTGACGGCAAACGAT-3′
		virC_R	5′-CGCCTCAAGGAGTGCCATTT-3′
virD1	GCA_000705135.2	virD1_F	5′-GCCGAAAATCCGACAATGGA-3′
		virD1_R	5′-CGTCTTTCAGCAGCGAGCAA-3′
virD2	GCA_000705135.2	virD2_F	5′-GAGTGGGCAGCCGAGATGTT-3′
		virD2_R	5′-ATCTTCAGCCAGCCGTGTCC-3′
virD4	GCA_000705135.2	virD4_F	5′-GTTGCGGGCATCCTTACCTG-3′
		virD4_R	5′-CGAAAATGCGCTGAGCCTCT-3′
virF	GCA_000705135.2	virF_F	5′-CGGCACGAAATCTTGGAAGC-3′
		virF_R	5′-CCGGGAATGACCGTATCGAA-3′
virG	GCA_000705135.2	virG_F	5′-TCGATGTCGTGGTCGTCGAT-3′
		virG_R	5′-TCCCAACTCGAGCGCAATAA-3′
virK	GCA_000705135.2	virK_F	5′-GCGGAGGCATTCGCATAGAT-3′
		virK_R	5′-CCGTCCAACCGGATTTGGTA-3′
Atu0972	NC_003062	Atu0972_F	5′-GTAGCCTGTTTGGCCGTTCG-3′
		Atu0972_R	5′-CCGTTTCCAGCGCCTTGTAT-3′
